# Impact of comorbidity on patient reported outcome measures for men with localised prostate cancer

**DOI:** 10.1186/s12894-026-02146-w

**Published:** 2026-04-18

**Authors:** Matthew G Parry, Anissa V Bailey, Thomas R Belin, David Elashoff, Claire Foster, Ian Graham, Christoph Kowalski, Lorna Kwan, Jeremy Millar, Nora Tabea Sibert, Sarah Weller, Holly Wilhalme, Mark S Litwin, Caroline M Moore, TrueNTH Global Registry

**Affiliations:** 1 Division of Surgical and Interventional Science, University College London, London, UK; 2 Departments of OBGYN, Surgery, & Urology, David Geffen School of Medicine, University of California, Los Angeles, CA USA; 3 Department of Biostatistics, Fielding School of Public Health, University of California, Los Angeles, CA USA; 4 Departments of Biostatistics and Medicine, University of California, Los Angeles, CA USA; 5 University of Southampton, Southampton, UK; 6 Ottawa Hospital Research Institute, Ottawa, Canada; 7 Department of Health Services Research, German Cancer Society, Berlin, Germany; 8 Monash University, Melbourne, Australia; 9 Oncological Health Services Research with a Focus on Digital Medicine, Department of Gynaecology and Obstetrics, University Hospital Düsseldorf, CIO ABCD, Heinrich-Heine University Düsseldorf, Düsseldorf, Germany; 10 Movember Institute of Men’s Health, Melbourne, Australia; 11 Department of Medicine Statistics Core, David Geffen School of Medicine, University of California, Los Angeles, CA USA; 12 University of California, Los Angeles, USA

**Keywords:** Prostate cancer, Patient-reported Outcome Measures, Functional outcomes, Comorbidities, International Registry

## Abstract

**Background:**

Urinary and sexual function are impacted by prostate cancer treatment but also by comorbidities. We aimed to report the impact of comorbidities on functional outcomes after prostate cancer treatment.

**Methods:**

We used patient reported EPIC-26, at baseline and 12 months, from a multicenter, prospective international cohort. Forest plots were constructed for urinary and sexual function, according to treatment, age and comorbidities (heart disease, hypertension and diabetes mellitus).

**Results:**

We identified 10,928 patients diagnosed with non-metastatic prostate cancer with complete PROMs at baseline and at 12 months. Urinary and sexual function at baseline varied with age and comorbidities, but the biggest impact on 12-month function was the treatment itself. Surgery had a greater impact on urinary function compared to radiotherapy, irrespective of comorbidities, but this was only observed for sexual function in those with no comorbidities. Men of younger age and those with no comorbidities had the best preservation of urinary and sexual function after treatment, but a floor effect was observed whereby those with better baseline sexual function reported larger post-treatment differences.

**Conclusions:**

The impact of age and comorbidity on functional outcomes after prostate cancer treatment is minimal when considered against the impact of the treatment itself.

**Supplementary Information:**

The online version contains supplementary material available at 10.1186/s12894-026-02146-w.

## Background

Multiple factors impact the outcomes and side effects that men experience in their prostate cancer care [1, 2]. We know that the experience of prostate cancer and its treatment can vary with disease factors and treatment differences as well as patient characteristics, such as age and health-related comorbidities. Both age and comorbidity play a role in treatment decision. However, previous studies have indicated that age may be more influential than having comorbidities in determining treatment receipt [3, 4]. The extent of the impact of comorbidities on patient-reported outcome measures (PROMs) in men having treatment for localised prostate cancer is less clear. A qualitative analysis evaluating how cancer survivors felt regarding their experiences with their diagnosis, treatment, life beyond cancer, and issues unrelated to living beyond cancer, found that many patients reported how their comorbidities added to their psychological, social, and physical difficulties after cancer treatment. These patients additionally reported that their cancer treatment had compounded the impact that their comorbidities had on their well-being [5].

PROMs are a tool for assessing the impact of prostate cancer treatment on symptoms, functional outcomes, and quality of life from the perspective of individual patients. They are valuable, alongside clinician-reported data, for a more comprehensive assessment of the impact of prostate cancer treatments to inform care [6]. They have been used effectively in healthcare performance assessment projects, such as the National Prostate Cancer Audit of England and Wales (NPCA), to capture variation in health outcomes between healthcare providers [7].

The TrueNTH Global Registry (TNGR), funded by Movember, was created to better understand why differences in prostate cancer care outcomes exist [8], in order to address modifiable factors and reduce unwarranted variation. The TNGR is an international registry that has collected clinical data and PROMs on men with localised and locally advanced prostate cancer from 16 countries across the world. Gathering this information in a large-scale registry provides a foundation for understanding how different treatments affect the quality of life of patients across a wide variety of settings.

The aim of this study is to provide stratified information to better inform both patients and clinicians about what to expect after treatment, and the impact that comorbidities have on functional outcomes. The TNGR offers a unique resource for providing international data in this area, enrolling men who have had treatment on routine care pathways rather than in strictly controlled trial environments.

## Methods

### True North Global Registry (TNGR)

The TNGR includes clinical data and PROMs of patients treated at one of the participating sites and who have non-metastatic prostate cancer. In some countries, a registry approach was used, and in others, patients were individually consented to participation and data collection in an ethics approved research study [9]. The TNGR participating countries are Australia, Austria, Canada, Czech Republic, Germany, Hong Kong, Italy, Luxembourg, Netherlands, New Zealand, Spain, Switzerland, United Kingdom (with sites from England, Northern Ireland, Scotland, and Wales) and the United States of America. To avoid the identification of the smaller providers from Austria, Czech Republic, Luxembourg, and Switzerland, data from these countries were pooled with data from Germany and labelled as ‘Central Europe’.

Patients had to give written informed consent to participate in all countries except for the centres in Australia, Canada, New Zealand and UK that use an opt-out-approach for clinical data and/or PROMs at some of their sites. Ethical approval was first given by the Monash University’s ethical committee and consecutively at each participating institution within the TNGR. The study was conducted in accordance with the ethical standards of the institutional research committees and the 2000 revision of the Declaration of Helsinki.

Clinical variables collected within the TNGR include age, comorbidities, prostate-specific antigen (PSA), and National Comprehensive Cancer Network (NCCN) risk classification [10]. Comorbidity was measured according to the self-administered comorbidity questionnaire in most countries and abstracted from the medical record in others [11]. PROMs were collected according to the abbreviated form of the Expanded Prostate Cancer Index Composite (EPIC-26) [12], and the additional three questions on the utilisation of sexual medicines which make up the International Consortium for Health Outcomes Measurement (ICHOM) within 90 days from their diagnosis biopsy and before starting their primary treatment.

For the purposes of this study, we focused on using two metrics from the EPIC-26 to provide proportions of men with erections sufficient for intercourse and those who were leak-free/pad-free. In this way we can provide comparisons across patient groups and treatments according to age and comorbidity, but also in a way that can be meaningful and informative to patients.

### Study population

**Fig. 1:**
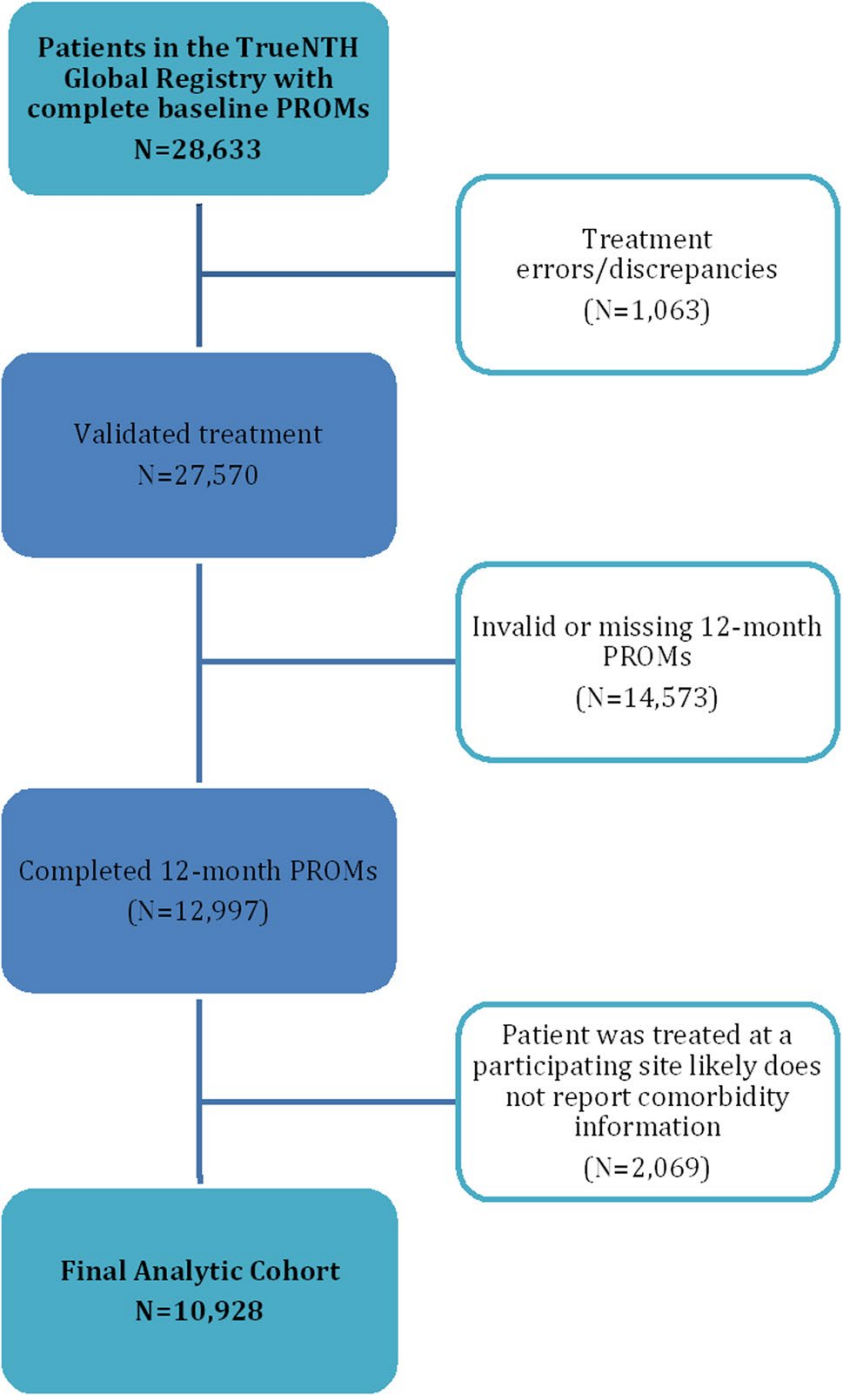
Patient flow chart

From the TNGR, we first identified 28,633 men diagnosed with non-metastatic prostate cancer who had complete data on baseline PROMs from January 2016 or later. Fig. 1 provides a flow diagram outlining the development of the analysis sample. Discrepancies in treatment information led to 1,063 men (3.7%) being excluded, yielding a sample of men who either underwent a single primary treatment (radical prostatectomy, radical radiotherapy or brachytherapy), or who commenced on active surveillance at diagnosis and did not transition to active treatment within the 12-month follow-up period. A further 14,573 men (50.8%) were not included because they did not have PROMs measured at 12 months, and the analysis sample was also limited to men who were treated at sites that reported comorbidities. If sites reported no men (either missing or marked ‘no’) with any comorbidities at all, we deemed that the sites did not report comorbidities. This criterion further excluded 2,069 men (7.2%), bringing the final analysis cohort to 10,928 and corresponding to 38.2% of the initial cohort.

### Statistical analysis

**Fig. 2:**
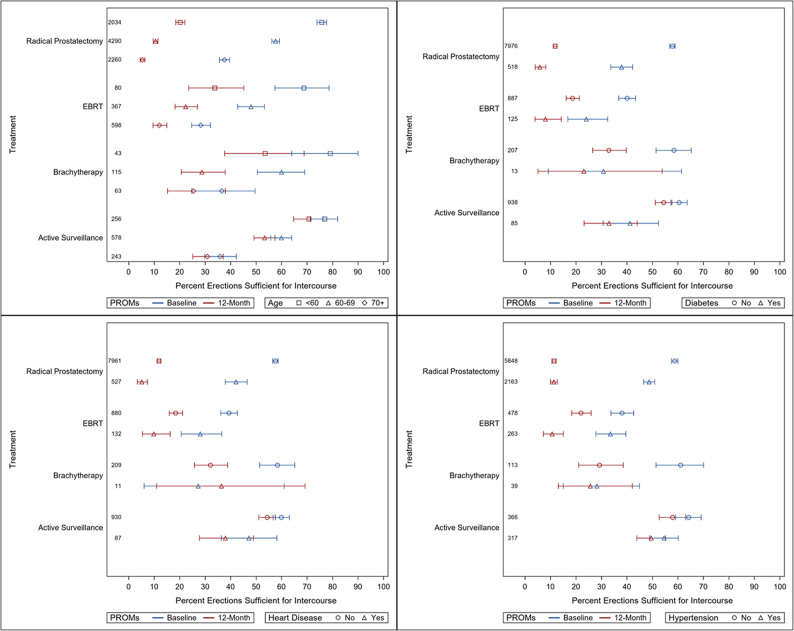
Forest plots for erectile function for each treatment group stratified by age and comorbidities

**Fig. 3:**
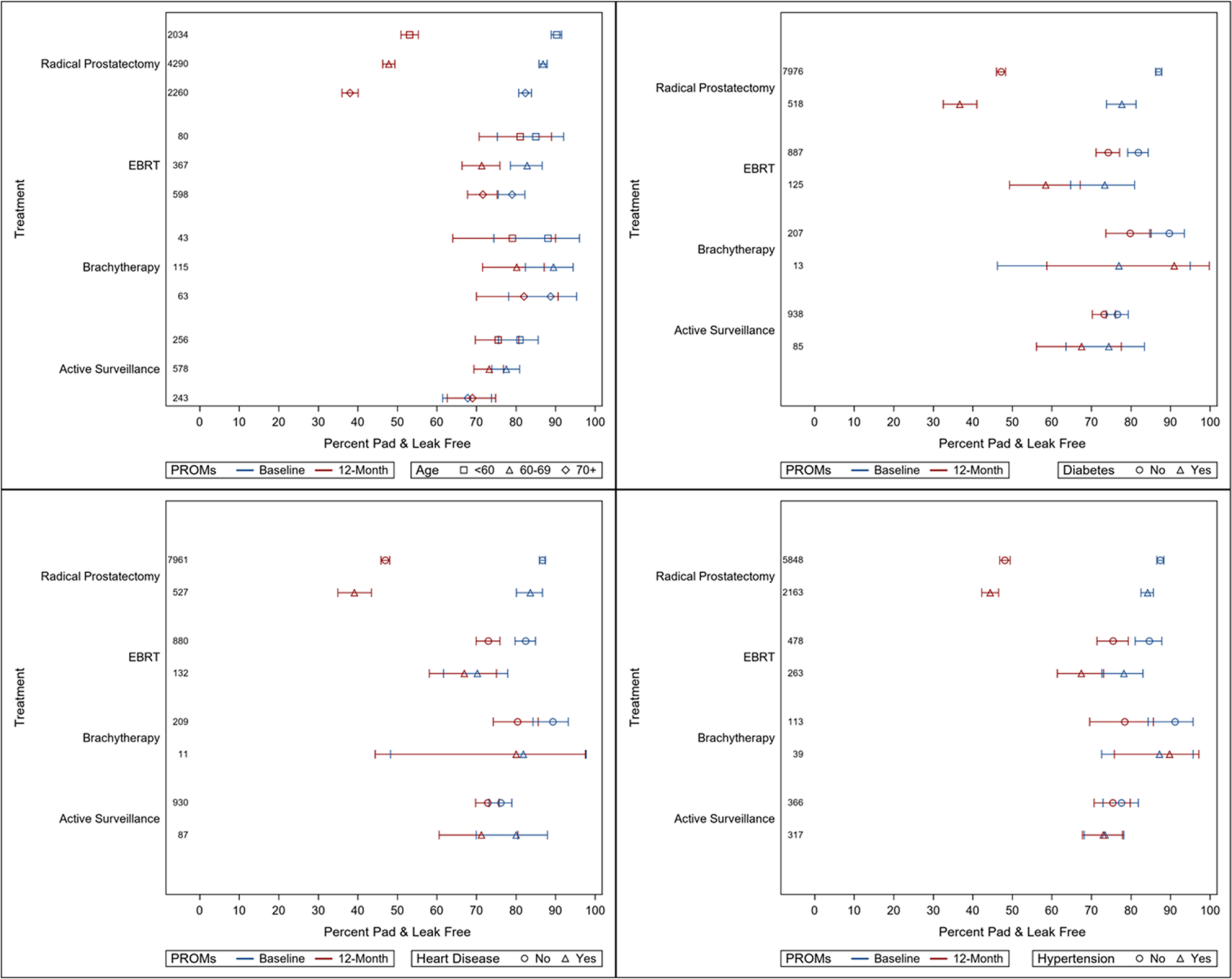
Forest plots for urinary continence for each treatment group stratified by age and comorbidities

Summary statistics of patient characteristics (Table 1) were reported per treatment. Forest plots were constructed for pad-free/leak-free status (Fig. 2) and proportion with erections sufficient for intercourse (with or without an aid) (Fig. 3) according to treatment (radical prostatectomy, radical radiotherapy, brachytherapy and active surveillance) and further stratified by age and the specific comorbidities of heart disease, hypertension, and diabetes mellitus (DM). These were reported as a proportion with a 95% confidence interval (CI). The comorbidities were selected based on their high frequency and known relevance to outcomes as per clinician expertise from the TrueNTH Project Team. For the purposes of this analysis, ‘no comorbidities’ was defined as not having any of the specific comorbidities of interest. Statistical analyses were conducted in SAS version 9.4 (SAS Institute, Cary, NC).

**Table 1:** Participant characteristics by treatment group

		Treatment			
	Total	Active Surveillance	Brachytherapy	Radical Radiotherapy	Radical Prostatectomy
	*N* = 10,928	*N* = 1077	*N* = 221	*N* = 1045	*N* = 8585
	n (%)	n (%)	n (%)	n (%)	n (%)
Age at Diagnosis					
< 55	906 (8.3%)	100 (9.3%)	17 (7.7%)	21 (2.0%)	768 (8.9%)
55–59	1507 (13.8%)	156 (14.5%)	26 (11.8%)	59 (5.6%)	1266 (14.7%)
60–64	2368 (21.7%)	265 (24.6%)	46 (20.8%)	126 (12.1%)	1931 (22.5%)
65–69	2982 (27.3%)	313 (29.1%)	69 (31.2%)	241 (23.1%)	2359 (27.5%)
70–74	2022 (18.5%)	142 (13.2%)	34 (15.4%)	267 (25.6%)	1579 (18.4%)
75+	1142 (10.5%)	101 (9.4%)	29 (13.1%)	331 (31.7%)	681 (7.9%)
Missing	1	0	0	0	1
Country					
Australia/New Zealand	856 (7.8%)	297 (27.6%)	25 (11.3%)	143 (13.7%)	391 (4.6%)
Canada	1111 (10.2%)	346 (32.1%)	47 (21.3%)	264 (25.3%)	454 (5.3%)
Central Europe	6987 (63.9%)	81 (7.5%)	84 (38.0%)	385 (36.8%)	6437 (75.0%)
Hong Kong	218 (2.0%)	37 (3.4%)	0 (0.0%)	77 (7.4%)	104 (1.2%)
Italy/Spain	705 (6.5%)	30 (2.8%)	43 (19.5%)	100 (9.6%)	532 (6.2%)
USA	747 (6.8%)	238 (22.1%)	0 (0.0%)	1 (0.1%)	508 (5.9%)
United Kingdom	304 (2.8%)	48 (4.5%)	22 (10.0%)	75 (7.2%)	159 (1.9%)
Year of Diagnosis					
2016	527 (4.8%)	6 (0.6%)	6 (2.7%)	25 (2.4%)	490 (5.7%)
2017	3553 (32.5%)	301 (27.9%)	100 (45.2%)	289 (27.7%)	2863 (33.3%)
2018	4963 (45.4%)	428 (39.7%)	99 (44.8%)	521 (49.9%)	3915 (45.6%)
2019	1876 (17.2%)	335 (31.1%)	16 (7.2%)	210 (20.1%)	1315 (15.3%)
2020	9 (0.1%)	7 (0.6%)	0 (0.0%)	0 (0.0%)	2 (0.0%)
NCCN Risk Group					
Low Risk	2247 (20.8%)	762 (75.9%)	97 (45.5%)	105 (10.1%)	1283 (15.0%)
Intermediate Risk	5876 (54.3%)	217 (21.6%)	113 (53.1%)	587 (56.7%)	4959 (57.9%)
(Very) High & Regional Risk	2692 (24.9%)	25 (2.5%)	3 (1.4%)	343 (33.1%)	2321 (27.1%)
Missing	113	73	8	10	22
Heart Disease					
No	9980 (92.9%)	930 (91.4%)	209 (95.0%)	880 (87.0%)	7961 (93.8%)
Yes	757 (7.1%)	87 (8.6%)	11 (5.0%)	132 (13.0%)	527 (6.2%)
Missing	191	60	1	33	97
Hypertension					
No	6805 (71.0%)	366 (53.6%)	113 (74.3%)	478 (64.5%)	5848 (73.0%)
Yes	2782 (29.0%)	317 (46.4%)	39 (25.7%)	263 (35.5%)	2163 (27.0%)
Missing	1341	394	69	304	574
Diabetes					
No	10,008 (93.1%)	938 (91.7%)	207 (94.1%)	887 (87.6%)	7976 (93.9%)
Yes	741 (6.9%)	85 (8.3%)	13 (5.9%)	125 (12.4%)	518 (6.1%)
Missing	179	54	1	33	91

## Results

The final cohort included 10,928 men diagnosed with non-metastatic prostate cancer with complete PROMs at baseline and at 12 months. 1,077 men underwent active surveillance, 221 underwent brachytherapy, 1,045 underwent radical radiotherapy and 8,585 underwent radical prostatectomy. Table 1 outlines the patient characteristics stratified by treatment group. Distribution across age groups was similar for the active surveillance, brachytherapy, and radical prostatectomy groups with the majority aged between 65 and 69 (27.3% to 31.2%), compared to the radical radiotherapy group who were older with one-third (31.7%) being 75 years or older. Men undergoing active surveillance or radical radiotherapy had a higher prevalence of comorbidities (heart disease, hypertension and DM) compared to men undergoing radical prostatectomy or brachytherapy.

### Prostate cancer risk stratification

More men having active surveillance had low risk disease (75.9%) compared to brachytherapy at 45.5%, radical radiotherapy at 10.1% and radical prostatectomy at 15.0%. The majority of those having treatment had intermediate risk disease or higher. 27.1% of men having radical prostatectomy, and 33.1% of men having radical radiotherapy, had higher risk disease at diagnosis.

### Active surveillance

Figures 2 and 3 show the forest plots for ‘Erections Sufficient for Intercourse’ and ‘Pad-Free/Leak-Free’ status at baseline and at 12 months. This is shown across different age groups and comorbidities.

As we would expect, baseline erectile function varied substantially across age groups. In the active surveillance cohort 77.0% of men < 60 years had erections sufficient for intercourse, compared to 59.9% for those aged 60–69 years and 35.8% for those of *≥* 70 years. Across all age groups, despite no curative treatment, there was some deterioration by 12 months (5–6%).

Baseline erectile function also varied with comorbidities. Around 64.7% of men without any comorbidities had erections sufficient for intercourse at baseline, which was higher than those with hypertension (54.6%), DM (41.2%) or heart disease (47.1%), dropping by 5% for those with no comorbidities and hypertension, and by 8% and 9% for those with DM and heart disease, respectively, over 12 months.

The proportion of men who were leak-free/pad-free at baseline was 81.0% in those aged < 60, 77.5% in those aged 60–69, and 67.8% in those *≥* 70. Fewer men were leak-free/pad-free at 12 months, with proportions reducing by 4–6% in the two former age groups and minimal change in the oldest group (in fact increasing by 1%).

The presence of comorbidities was not associated with significant differences in leak-free/pad-free status at baseline in men on active surveillance (78.1% for men with no comorbidities, 74.4% with DM, 73.4% with hypertension, and 80.0% for those with heart disease). Similar to age, fewer men with comorbidities were leak-free/pad-free at 12 months. In those with hypertension, less than 1% lost leak-free/pad-free status, while 8.7% and 6.9% of those with heart disease and DM lost their leak-free/pad-free status, respectively.

### Radical prostatectomy

Men having radical prostatectomy had a lower median age than other groups (65.0 years vs. 66.0 years for the overall cohort).

At baseline we saw a difference in erections sufficient for intercourse across age groups (75.8% in men under 60, 57.6% aged 60–69, and 37.6% for those aged *≥* 70). There was a significant reduction across all age groups at 12 months - only 20.2% of those < 60, 10.5% of those aged 60–69 and 5.4% of those aged *≥* 70 reported having erections sufficient for intercourse.

When we look at the subgroup of men with erections sufficient for intercourse at baseline (Supplementary Table 1) we see that at 12 months this varied according to age and had notably reduced to 24.7% in those < 60, 14.7% in those aged 60–69 and 8.4% in those aged *≥*70 years.

Sexual function also varied with the presence of comorbidities. In the radical prostatectomy cohort, for those without any comorbidities, 59.7% of men had erections sufficient for intercourse at baseline, dropping to 11.7% at 12 months. This was lower at baseline and 12 months for those with DM (37.8% to 5.8%), hypertension (48.7% to 11.3%), and heart disease (42.1% to 5.1%). For a subset of those < 60 with no comorbidities (representing the ‘ideal surgical candidate’), the respective figures were 78.1% at baseline dropping to 19.3%, compared to 67.9% at baseline dropping to 21.5% for those with at least one comorbidity of interest.

Leak-free/pad-free status varied with age, with 90.3% of men aged < 60 years being leak-free/pad-free at baseline, compared to 86.9% for those aged 60–69 years, and 82.3% for those aged *≥*70 years. By 12 months these proportions fell to 53.1% in those under 60 years, 47.8% for those aged 60–69 years, and 38.0% for those aged *≥* 70 years.

Leak-free/pad-free status also varied with comorbidities at baseline and 12 months. At baseline, 87.8% of men without any comorbidities were leak-free/pad-free, which dropped to 48.5% at 12 months after radical prostatectomy. These figures were 77.7% to 36.7% in men with DM, 84.2% to 44.4% for those with hypertension, and 83.6% to 39.1% for those with heart disease. For a subset of those < 60 years with no comorbidities (representing the ‘ideal surgical candidate’), the respective figures were 91.6% at baseline dropping to 55.2%, compared to 87.5% at baseline dropping to 50.5% for those with at least one comorbidity of interest.

### Radical radiotherapy

Men having radical radiotherapy had a higher median age than other groups (71.0 years), and more men with higher risk disease (33.6%). Baseline erectile function was lower for this group across all age groups, with 68.8%, 48.0% and 28.3% of men in age groups < 60 years, 60–69 years, and *≥* 70 years, respectively. These figures dropped at 12 months, but to a lesser extent than those in the radical prostatectomy group, with erections sufficient for intercourse seen in 33.8% of those aged < 60 years, 22.3% for those aged 60–69 years and 12.0% for those aged *≥* 70 years.

As expected, function also varied with the presence of comorbidities with erections sufficient for intercourse at baseline in 39.5% of men without comorbidities, 24.0% for those with DM, 33.5% for those with hypertension, and 28.0% for those with heart disease. Proportions fell at 12 months to 23.3% for those with no comorbidities, 8.0% for those with DM, 10.6% for those with hypertension and 9.8% for those with heart disease.

Similar but small age-related differences in leak-free/pad-free status at baseline were seen, with 84.9% of those aged < 60 years being leak-free/pad-free, compared to 83.0% in those aged 60–69 years and 78.0% in those aged *≥* 70 years. This reduced at 12 months after radical radiotherapy, but to a lesser extent than those having radical prostatectomy, to 81.2% for those < 60 years, 71.9% for those aged 60–69 years and 70.9% for those aged *≥* 70 years.

Differences were seen according to comorbidity status with 85.2% of men without comorbidities being leak-free/pad-free at baseline, compared to 73.4% for men with DM, 78.2% with hypertension, and 70.2% for men with heart disease. These figures were lower at 12 months with the greatest reduction in men with DM (15.0%), compared to 10.7% in those with hypertension, and 3.3% in those with heart disease.

### Brachytherapy

Due to low patient numbers and wide confidence intervals, the interpretation of the forest plots was difficult for men who underwent brachytherapy, especially for the comorbidity groups. General trends observed were similar to those shown in the other treatment groups in that younger age groups had better baseline function, as did those without DM, heart disease and hypertension.

79.1% of men aged under 60 years had erections sufficient for intercourse at baseline which dropped to 53.5% at 12 months after brachytherapy (25.6% change), compared with 60.0% to 28.7% for men aged 60–69 years (31.3% change), and 36.5% to 25.4% for men aged 70 years and over (11.1% change). Irrespective of age group, brachytherapy decreased the proportion of men who were leak-free/pad-free at baseline (87.0-88.7%) by approximately 10% in those < 60 years and 60–69 years but only 6.7% in those aged *≥* 70 years at 12 months.

Brachytherapy appears to have a similar impact on erectile function and urinary continence compared to radical radiotherapy. 61.7% of men without comorbidities had erections sufficient for intercourse at baseline which dropped to 29.0% at 12 months after brachytherapy (32.7% change), and 91.6% of men without comorbidities were leak-free/pad-free at baseline which dropped to 78.3% at 12 months after brachytherapy (13.3% change).

## Discussion

Urinary and sexual function in men with localised prostate cancer varies with both age and the presence of comorbidities, both at baseline and after 12 months. In our cohort, the biggest change in urinary and sexual function was seen after treatment, with radical prostatectomy having a greater impact on these than radical radiotherapy, consistent with previous literature. A systematic review found that sexual dysfunction and urinary leakage was worse in localized prostate cancer patients treated with radical prostatectomy than those treated with radical radiotherapy or brachytherapy [13]. The ProtecT trial yielded similar results but noted that there were differences between treatments with longer follow-up [14].

There was a trend between age and comorbidity leading to a higher impact of treatment, with men of younger age and no comorbidities having better preservation of function than older men with comorbidities. A previous study evaluating advanced prostate cancer patients found that comorbidity burden was negatively associated with PROMs, independent of medical and sociodemographic factors [15]. Additionally, their review of other studies demonstrated that higher numbers of comorbidities were associated with poorer physical health [15].

It is well documented that comorbidities such as heart disease and DM, in combination with advancing age, are risk factors for erectile dysfunction in the general population [16, 17]. We observed this in our active surveillance cohort where we found differences in baseline sexual function across age and comorbidity groups, as expected, but the decline in function at one year was consistently 5–10% in the proportion of those with erections sufficient for intercourse, irrespective of age or comorbidity. This was not observed for the treatment groups whereby those with the better baseline sexual function had the more marked drops in sexual function at one year (younger patients, irrespective of treatment, and surgical patients with no comorbidities) - likely a floor effect in which those with better function at baseline had more to lose. This was particularly noted for the ‘ideal surgical candidate’ whereby those < 60 years with no comorbidities had better function at baseline compared to those with comorbidities, but the difference was lost by 12 months after surgery.

For urinary continence there was much less variation observed across age and comorbidity groups at baseline compared to sexual function, with leak-free/pad-free proportions mostly above 80%. We also observed quite consistent changes in urinary continence irrespective of age or comorbidity (up to 15% for radical radiotherapy and brachytherapy patients, and between 37% and 45% for surgical patients), indicating that age and comorbidity have a limited impact in post-treatment urinary continence versus the type of treatment. This is somewhat contrary to some previous studies reporting that certain clinical variables (i.e., age, PSA, adjuvant androgen deprivation therapy, and tumour volume) greatly influenced post-treatment function [13]. In addition, the impact of radiation (radical radiotherapy or brachytherapy) on urinary incontinence is only modestly higher than that experienced by men on active surveillance (~ 5% decline in the proportion who are leak-free/pad-free).

The major strengths of our study were the inclusion of a large number of patients representing a ‘real world’ contemporary population across multiple countries and the use of relevant, patient-specific measures of erectile function and urinary continence. An important limitation is the large number of exclusions made due to treatment errors/discrepancies, invalid/missing 12-month PROMs or some participating sites not reporting comorbidity information (17,705 patients, 71.8%). This could likely lead to a selection bias in our final patient cohort.

Further to this, overall missing data of comorbidity status was modest and ranged from 7% to 17%, except for data on hypertension which was missing for 42% of the active surveillance group, 35% of the radical radiotherapy group and 36% of the brachytherapy group. Again, this is a potential source of selection bias but we would not expect the patients with missing data to be substantially different to those with complete data, and so the impact on interpretation is likely to be minimal. We also acknowledge that those having radical prostatectomy make up the majority of the cohort at nearly 80%, three quarters of whom were exclusively from Central Europe, for which we understand the selection bias implications and the additional benefit in including patients across all treatment modalities from the same centres, during the same time period. This said, of the included patients, there is no reason to believe that these patients would differ substantially from the general population of prostate cancer patients, and patients in the active surveillance or treatment groups would still be representative of ‘real world’ patients.

Our focus in the analysis on individuals with complete data on baseline PROMs, baseline comorbidities, and 12-month PROMs is a limitation that deserves attention. While complete-case estimates of population prevalences can incur substantial selection bias when a sizable proportion of patients are omitted from the analysis, inferences are not subject to bias when inclusion for analysis depends only on covariates and not residually on outcomes [18]. Here, we do not attempt to describe population prevalences of various complications, rather our aim is to compare frequencies of complications across population subgroups in ways that can inform clinical practice and patients. It is conceivable that patients who do not have recorded 12-month measurements might have different outcome profiles from those who do. Accordingly, estimated proportions of various outcomes should be understood to have the potential to depend on the mechanism that gave rise to certain individuals being assessed at 12 months and others not. That said, to the extent that a process yielded differences in outcome distributions between those who had 12-month measurements and those who did not, it stands to reason that such a process would have similar impacts on subgroups characterized by primary treatments for prostate cancer and comorbidities, implying that comparisons across primary-treatment arms and comorbidity subgroups would still yield insights relevant to clinical practice.

It is important to highlight that we reported outcomes in terms of ‘erections sufficient for intercourse’ and ‘leak-free/pad-free’ status rather than EPIC-26 scores, in order for results to be most relevant for patient interpretation. This is therefore at the expense of accounting for quality-of-life implications which are also captured within the EPIC-26. Given men could feasibly have poor erectile quality but not deem it to be a ‘problem’ for their sexual function, it is important to be mindful that our outcomes could over-estimate ‘poor’ function, but given our main objective was to assess the association between comorbidity and functional outcomes we do not feel this would affect the interpretation of our results. Further to this, we only report outcomes at 12 months and fully appreciate that functional outcomes can still improve after this time point for surgical patients and upon cessation of androgen deprivation therapy for the men receiving radiotherapy. We do not have information about concurrent androgen deprivation therapy but would expect that many patients will be experiencing symptoms related to recent or ongoing androgen deprivation therapy at the time of their final questionnaire completion, and to interpret our results as such.

A further potential limitation would be the variation in data collection across the countries included in the study. In some of the sites in three regions (i.e., Canada, Central Europe, and the United States), where it was not explicitly stated that a comorbidity was present, it was assumed to be absent. This potential misclassification could lead to an underestimation of the effect of the comorbidity. In addition, there will always be variation between countries in how treatments are delivered and we would expect that these differences would lead to variation in post-treatment outcomes. For example, with regards to the use of nerve-sparing surgery or the specific fractionation used for radical radiotherapy, which are both associated with differences in functional outcomes. It has already been shown in a prior publication from the TNGR that country is a relevant predictor for incontinence and sexual function scores after surgery and it is clear that functional outcomes after prostate cancer treatment is multi-factorial, based on wide ranging pre-, peri- and post-operative factors [19].

In conclusion, the impact of age and comorbidity on functional outcomes after treatment for prostate cancer is minimal when considered against the impact of the treatment itself. Age and specific comorbidities are important for treatment selection and decision-making but should not individually preclude treatment for prostate cancer, where a holistic approach should be used. Patients should be aware that the biggest impact on post-treatment functional status is the treatment itself.

## Supplementary Information

Supplementary Material 1.

## Data Availability

AVB had full access to all the data in the study and takes responsibility for the integrity of the data and accuracy of the data analysis. The TNGR is collated at Monash University and is therefore not publicly available.
