# Supplementary material for: Impact of comorbidity on patient reported outcome measures for men with localised prostate cancer
Source: BMC Urol. 2026 Apr 18;26:135. doi: 10.1186/s12894-026-02146-w (PMC13231530; doi:10.1186/s12894-026-02146-w)
Supplement: Supplementary file 1 — Supplementary Material 1. [file 12894_2026_2146_MOESM1_ESM.docx]

**Supplementary Table 1.** Proportions of men with erections sufficient for intercourse at 12 months in those with normal erectile function at baseline stratified by treatment.

|  | Treatment | | | |
| --- | --- | --- | --- | --- |
|  | Active Surveillance  N=583 | Brachytherapy  N=122 | Radial Radiotherapy  N=357 | Radical Prostatectomy  N=4701 |
|  | *n / N (%)* | *n / N (%)* | *n / N (%)* | *n / N (%)* |
| **Aged <60** (N=1792) | 156/184 (84.8) | 22/34 (64.7) | 23/52 (44.2) | 376/1522 (24.7) |
| **Aged 60-69** (N=2946) | 244/325 (75.1) | 23/66 (34.9) | 65/165 (39.4) | 351/2390 (14.7) |
| **Aged 70+** (N=1025) | 45/74 (60.8) | 12/22 (54.6) | 39/140 (27.9) | 66/789 (8.4) |
| **No Comorbidities** (N=3567) | 162/202 (80.2) | 27/64 (42.2) | 74/150 (49.3) | 527/3151 (16.7) |
| **Heart Disease** (N=285) | 25/37 (67.6) | 1/3 (33.3) | 8/34 (23.5) | 18/211 (8.5) |
| **Hypertension** (N=1266) | 122/161 (75.8) | 6/11 (54.6) | 19/78 (24.4) | 181/1016 (17.8) |
| **Diabetes** (N=249) | 19/31 (61.3) | 2/4 (50.0) | 2/27 (7.4) | 20/187 (75.1) |

**Supplementary Table 2.** Proportions of men pad-free/leak-free at 12 months in those with normal urinary continence at baseline stratified by treatment.

|  | Treatment | | | |
| --- | --- | --- | --- | --- |
|  | Active Surveillance  N=800 | Brachytherapy  N=194 | Radial Radiotherapy  N=829 | Radical Prostatectomy  N=7325 |
|  | *n / N (%)* | *n / N (%)* | *n / N (%)* | *n / N (%)* |
| **Aged <60** (N=2127) | 174/204 (85.3) | 30/37 (81.1) | 59/68 (86.8) | 1010/1818 (55.6) |
| **Aged 60-69** (N=4518) | 359/438 (82.0) | 83/102 (81.4) | 227/299 (75.9) | 1876/3679 (51.0) |
| **Aged 70+** (N=2503) | 125/158 (79.1) | 47/55 (85.5) | 371/462 (80.3) | 774/1828 (42.3) |
| **No Comorbidities** (N=5452) | 213/249 (85.5) | 81/98 (82.7) | 302/362 (83.4) | 2444/4743 (51.5) |
| **Heart Disease** (N=602) | 56/68 (82.4) | 7/9 (77.8) | 67/92 (72.8) | 188/433 (43.4) |
| **Hypertension** (N=2273) | 199/229 (86.9) | 31/34 (91.2) | 155/204 (76.0) | 869/1806 (48.1) |
| **Diabetes** (N=559) | 46/61 (75.4) | 9/10 (90.0) | 64/91 (70.3) | 176/397 (44.3) |

**Supplementary Table 3.** Proportions of men with erections sufficient for intercourse at baseline and 12 months according to treatment, stratified by age and comorbidities (proportions underpinning figures 2 and 3).

|  | Treatment | | | | | | | |
| --- | --- | --- | --- | --- | --- | --- | --- | --- |
|  | Active Surveillance | | Brachytherapy | | Radial Radiotherapy | | Radical Prostatectomy | |
|  | Baseline | 12-Months | Baseline | 12-Months | Baseline | 12-Months | Baseline | 12-Month |
| *Erections Sufficient for Intercourse, % (95% CI)* | | | | | | | | |
| Age | | | | | | | | |
| <60 | 77.0%  (71.3%, 82.0%) | 70.7%  (64.7%, 76.2%) | 79.1%  (64.0%, 90.0%) | 53.5%  (37.7%, 68.8%) | 68.8%  (57.4%, 78.7%) | 33.8%  (23.6%, 45.2%) | 75.8%  (73.8%, 77.6%) | 20.2%  (18.5%, 22.0%) |
| 60-69 | 59.9%  (55.7%, 63.9%) | 53.3%  (49.1%, 57.4%) | 60.0%  (50.4%, 69.0%) | 28.7%  (20.6%, 37.9%) | 48.0%  (42.7%, 53.2%) | 22.3%  (18.2%, 27.0%) | 57.6%  (56.2%, 59.1%) | 10.5%  (9.6%, 11.5%) |
| 70+ | 35.8%  (29.8%, 42.2%) | 30.9%  (25.1%, 37.1%) | 36.5%  (24.7%, 49.6%) | 25.4%  (15.3%, 37.9%) | 28.3%  (24.7%, 32.1%) | 12.0%  (9.5%, 14.9%) | 37.6%  (35.6%, 39.6%) | 5.4%  (4.5%, 6.4%) |
| Comorbidities of Interest | | | | | | | | |
| No | 64.7%  (59.3%, 69.9%) | 59.2%  (53.7%, 64.6%) | 61.7%  (51.8%, 70.9%) | 29.0%  (20.6%, 38.5%) | 39.5%  (34.8%, 44.3%) | 23.4%  (19.4%, 27.7%) | 59.7%  (58.3%, 61.0%) | 11.7%  (10.8%, 12.6%) |
| Yes | 53.4%  (48.4%, 58.4%) | 48.1%  (43.1%, 53.2%) | 32.1%  (19.9%, 46.3%) | 28.3%  (16.8%, 42.3%) | 31.1%  (26.5%, 36.0%) | 9.9%  (7.1%, 13.4%) | 48.3%  (46.3%, 50.2%) | 10.4%  (9.3%, 11.7%) |
| Heart Disease | | | | | | | | |
| No | 59.9%  (56.7%, 63.1%) | 54.3%  (51.0%, 57.5%) | 58.4%  (51.4%, 65.1%) | 32.1%  (25.8%, 38.8%) | 39.3%  (36.1%, 42.6%) | 18.4%  (15.9%, 21.1%) | 57.6%  (56.5%, 58.7%) | 11.8%  (11.1%, 12.6%) |
| Yes | 47.1%  (36.3%, 58.1%) | 37.9%  (27.7%, 49.0%) | 27.3%  (6.0%, 61.0%) | 36.4%  (10.9%, 69.2%) | 28.0%  (20.6%, 36.5%) | 9.8%  (5.3%, 16.3%) | 42.1%  (37.9%, 46.5%) | 5.1%  (3.4%, 7.4%) |
| Hypertension | | | | | | | | |
| No | 64.2%  (59.1%, 69.1%) | 57.9%  (52.7%, 63.0%) | 61.1%  (51.4%, 70.1%) | 29.2%  (21.0%, 38.5%) | 38.1%  (33.7%, 42.6%) | 22.0%  (18.3%, 26.0%) | 58.7%  (57.4%, 60.0%) | 11.3%  (10.5%, 12.2%) |
| Yes | 54.6%  (48.9%, 60.1%) | 49.5%  (43.9%, 55.2%) | 28.2%  (15.0%, 44.9%) | 25.6%  (13.0%, 42.1%) | 33.5%  (27.8%, 39.5%) | 10.6%  (7.2%, 15.0%) | 48.7%  (46.6%, 50.9%) | 11.3%  (10.0%, 12.7%) |
| Diabetes | | | | | | | | |
| No | 60.4%  (57.2%, 63.6%) | 54.4%  (51.1%, 57.6%) | 58.5%  (51.4%, 65.2%) | 32.9%  (26.5%, 39.7%) | 40.0%  (36.8%, 43.3%) | 18.6%  (16.1%, 21.3%) | 57.8%  (56.7%, 58.9%) | 11.8%  (11.1%, 12.5%) |
| Yes | 41.2%  (30.6%, 52.4%) | 32.9%  (23.1%, 44.0%) | 30.8%  (9.1%, 61.4%) | 23.1%  5.0%, 53.8%) | 24.0%  (16.8%, 32.5%) | 8.0%  (3.9%, 14.2%) | 37.8%  (33.6%, 42.2%) | 5.8%  (3.9%, 8.2%) |

**Supplementary Table 4.** Proportions of men leak-free/pad-free at baseline and 12 months according to treatment, stratified by age and comorbidities (proportions underpinning figures 2 and 3).

|  | Treatment | | | | | | | |
| --- | --- | --- | --- | --- | --- | --- | --- | --- |
|  | Active Surveillance | | Brachytherapy | | Radial Radiotherapy | | Radical Prostatectomy | |
|  | Baseline | 12-Months | Baseline | 12-Months | Baseline | 12-Months | Baseline | 12-Month |
| *Leak-Free/Pad-Free, % (95% CI)* | | | | | | | | |
| Age | | | | | | | | |
| <60 | 81.0%  (75.6%, 85.6%) | 75.5%  (69.7%, 80.7%) | 88.1%  (74.4%, 96.0%) | 79.1%  (64.0%, 90.0%) | 85.0%  (75.3%, 92.0%) | 81.0%  (70.6%, 89.0%) | 90.3%  (88.9%, 91.5%) | 53.1%  (50.9%, 55.3%) |
| 60-69 | 77.5%  (73.9%, 80.9%) | 73.2%  (69.3%, 76.8%) | 89.5%  (82.3%, 94.4%) | 80.2%  (71.5%, 87.1%) | 82.8%  (78.5%, 86.6%) | 71.3%  (66.3%, 75.9%) | 86.9%  (85.8%, 87.9%) | 47.8%  (46.3%, 49.3%) |
| 70+ | 67.8%  (61.4%, 73.8%) | 69.0%  (62.6%, 74.9%) | 88.7%  (78.1%, 95.3%) | 82.0%  (70.0%, 90.6%) | 79.0%  (75.4%, 82.2%) | 71.6%  67.8%, 75.2%) | 82.3%  (80.7%, 83.9%) | 38.0%  (36.0%, 40.0%) |
| Comorbidities of Interest | | | | | | | | |
| No | 78.1%  (73.1%, 82.5%) | 76.2%  (71.2%, 80.8%) | 91.6%  (84.6%, 96.1%) | 78.3%  (69.2%, 85.7%) | 85.2%  (81.4%, 88.4%) | 76.1%  (71.8%, 80.1%) | 87.8%  (86.9%, 88.7%) | 48.5%  (47.2%, 49.9%) |
| Yes | 74.6%  (70.0%, 78.9%) | 71.5%  (66.7%, 75.9%) | 84.9%  (72.4%, 93.3%) | 86.0%  (73.3%, 94.2%) | 77.4%  (72.8%, 81.5%) | 66.0%  (61.0%, 70.8%) | 83.6%  (82.1%, 85.0%) | 43.7%  (41.8%, 45.6%) |
| Heart Disease | | | | | | | | |
| No | 76.2%  (73.2%, 78.9%) | 72.8%  (69.8%, 75.7%) | 89.3%  (84.3%, 93.2%) | 80.4%  (74.3%, 85.6%) | 82.4%  (79.7%, 84.9%) | 73.0%  (69.9%, 75.9%) | 86.7%  (85.9%, 87.4%) | 46.9%  (45.8%, 48.0%) |
| Yes | 80.0%  (69.9%, 87.9%) | 71.3%  (60.6%, 80.5%) | 81.8%  (48.2%, 97.7%) | 80.0%  (44.4%, 97.5%) | 70.2%  (61.6%, 77.9%) | 66.9%  (58.0%, 75.0%) | 83.6%  (80.1%, 86.7%) | 39.1%  (34.9%, 43.4%) |
| Hypertension | | | | | | | | |
| No | 77.6%  (72.9%, 81.8%) | 75.4%  (70.6%, 79.8%) | 91.2%  (84.3%, 95.7%) | 78.4%  (69.6%, 85.6%) | 84.6%  (81.1%, 87.8%) | 75.5%  (71.4%, 79.3%) | 87.4%  (86.6%, 88.3%) | 48.1%  (46.8%, 49.4%) |
| Yes | 73.4%  (68.1%, 78.2%) | 73.0%  (67.8%, 77.8%) | 87.2%  (72.6%, 95.7%) | 89.7%  (75.8%, 97.1%) | 78.2%  (72.7%, 83.0%) | 67.4%  (61.4%, 73.1%) | 84.2%  (82.5%, 85.7%) | 44.4%  (42.2%, 46.5%) |
| Diabetes | | | | | | | | |
| No | 76.6%  (73.7%, 79.3%) | 73.2%  (70.2%, 76.0%) | 89.7%  (84.7%, 93.5%) | 79.8%  (73.6%, 85.1%) | 81.9%  (79.1%, 84.4%) | 74.2%  (71.2%, 77.1%) | 87.0%  (86.2%, 87.7%) | 47.1%  (46.0%, 48.2%) |
| Yes | 74.4%  (63.6%, 83.4%) | 67.5%  (56.1%, 77.6%) | 76.9%  (46.2%, 95.0%) | 90.9%  (58.7%, 99.8%) | 73.4%  (64.7%, 80.9%) | 58.4%  (49.2%, 67.1%) | 77.7%  (73.8%, 81.2%) | 36.7%  (32.5%, 41.0%) |
